# Brainstem Infarction in Immunodeficiency Identified as Adenosine Deaminase 2 Deficiency: Case Report

**DOI:** 10.1007/s10875-023-01526-3

**Published:** 2023-06-12

**Authors:** Christoph Oster, Benjamin Stolte, Livia Asan, Refik Pul, Stephan Klebe, Martin Köhrmann, Katharina Breuckmann, Christoph Rischpler, Cornelius Deuschl, Sebastian Dolff, Christoph Kleinschnitz, Tim Hagenacker

**Affiliations:** 1 Department of Neurology and Center for Translational Neuro- and Behavioral Sciences (C-TNBS), University Hospital Essen, University of Duisburg-Essen, Essen, Germany; 2 Institute for Diagnostic and Interventional Radiology and Neuroradiology, University Hospital Essen, University of Duisburg-Essen, Essen, Germany; 3 Department of Nuclear Medicine, University Hospital Essen, University of Duisburg-Essen, Essen, Germany; 4 Department of Infectious Diseases, University Hospital Essen, University of Duisburg-Essen, Essen, Germany

**Keywords:** Ataxia, CVID, DADA2, Immunoglobulins, Immunosuppression, Stroke

## Abstract

**Purpose:**

We present the case of a 24-year-old male with CNS granulomatosis due to an immunodeficiency syndrome which was identified as deficiency of adenosine deaminase 2 (DADA2) as a cause of brainstem infarction.

**Methods:**

Case report and detailed description of the clinical course of diagnosis and treatment.

**Case:**

The patient’s medical history consisted of an unknown immunodeficiency syndrome. Based on former findings, common variable immunodeficiency (CVID) was diagnosed. The patient suffered from three consecutive brainstem strokes of unknown etiology within 3 years. An MRI scan detected gadolinium-enhancing, granulomatous-suspect lesions in the interpeduncular cistern, temporal lobe, and tegmentum. Laboratory analysis was compatible with CVID, with leukopenia and immunoglobulin deficiency. Because granulomatous CNS inflammation was suspected, the patient received methylprednisolone immunosuppressive therapy, which led to partially regressive MRI lesions. However, in contrast to imaging, the patient showed a progressive cerebellar syndrome, indicating plasma exchange therapy and immunoglobulin treatment, which led to rapid symptom amelioration. After a relapse and a further stroke, expanded analysis confirmed DADA2 (and not CVID) as the inflammatory cause for recurrent stroke. After starting the therapy with immunoglobulins and adalimumab, no further strokes occurred.

**Conclusion:**

We present the case of a young adult with diagnosis of DADA2 as a cause for recurrent strokes due to vasculitis. This stroke etiology is rare but should be considered as a cause of recurrent stroke of unknown origin in young patients to avoid a disabling disease course by disease-specific treatment options.

## Introduction

We present the case of a 24-year-old male with CNS granulomatosis due to an immunodeficiency syndrome which was identified as deficiency of adenosine deaminase 2 (DADA2) as a cause of cerebellar syndrome and brainstem infarction.

## Methods

This is a case report and a detailed description of the clinical course of diagnosis and treatment of DADA2. Local ethics vote is not needed because patient signed informed consent and no study was performed.

## Case Description

**Fig. 1:**
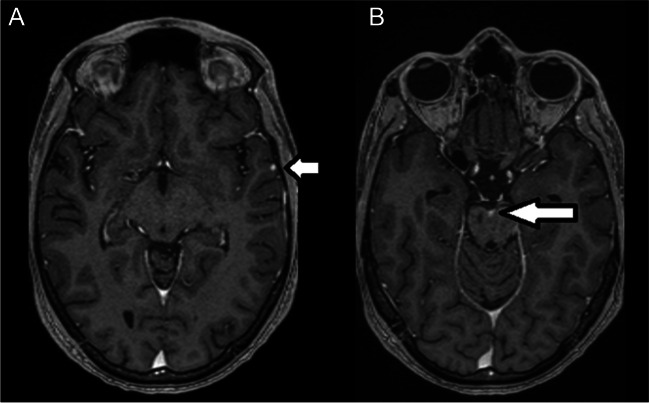
Gadolinium-enhanced MRI T1 (MP-RAGE) showed a focal-enhancing lesion cortical in the left temporal lobe (**A**) and interpeduncular cistern (**B**)

In July 2021, the patient was admitted to our department after a stroke, under suspicion of vasculitis. The patient was suspected to suffer from CVID, with predominant reduction of T-lymphocytes, immunoglobulin (Ig)A, and IgM, which had been diagnosed during childhood. He suffered his first ischemic stroke of unknown etiology in August 2018 (age: 21), followed by another in March 2021 (age: 23). The patient suffered from dizziness, double vision and dysarthria at that time. During childhood, Kawasaki disease was suspected after episodes of recurrent fever and arthralgia. The patient’s medical history consisted of splenomegaly, pancreatic lipomatosis, hypothyroidism, and steatosis hepatis. In June 2021, the patient suffered from paresthesia in the right side of the face, downbeat-nystagmus, nystagmus in fixation, internuclear ophthalmoplegia, and fall inclination to the right. MRI brain imaging revealed a paramedian brainstem infarction with nodular gadolinium enhancement in the interpeduncular cistern, left temporal lobe, and tegmentum. The lesions were suspected to be of granulomatous origin (Fig. 1A, B). Only the brainstem lesion was diagnosed as infarction due to acute onset of neurological deficits (explained by acute brainstem pathology), typical localization and signal restriction in diffusion weighted MRI sequences. The other suspect lesions’ distribution pattern and appearance convinced the adjudged radiologists to be from inflammatory source. Lesion biopsies could not be feasibly obtained due to the small size and a risk-prone neurosurgical access route.

Over the following days, the neurologic symptoms worsened, with double vision, dizziness, encephalopathy, and fever. Laboratory analysis detected an increased erythrocyte sedimentation rate (ESR, 43 mm after 1 h (norm value less than 10 mm per hour) and 77 mm after 2 h), elevated white blood count (WBC), and increased C-reactive protein (CRP, 6.7 mG per dL, normal value < 0.5 mG per dL).

The patient was referred to our department. The WBC revealed a massive decrease in B- and T-cells, including subpopulations (Table 1). IgM and IgA levels were below the detection rate. In IgG subclass analysis, a selective reduction of IgG2 and IgG4 with normal IgG1 and IgG3 levels was detected. No signs of systemic vasculitis, paraneoplastic syndrome, or sarcoidosis were found in laboratory chemistry. Antinuclear antibodies, monoclonal antibodies, soluble interleukin-2 receptor, and the anti-neutrophil cytoplasmic antibodies in serum were negative. Lupus anticoagulant was marginally increased (under enoxaparin treatment). Alanine and aspartate aminotranferases showed normal values.

**Table 1:** Distinct lab parameters

Parameter	Measured value	Normal values
Leucocytes (per nL)	3.54	3.6–9.2
Lymphocytes (per nL)	0.51	1.0–3.4
B-cells (CD19 +) (per μL)	72	132–339
T-cells (CD3 +) (per μL)	308	1105–1978
T-helper cells (CD3 + CD4 +) (per μL)	153	604–1188
T-suppressor-cells (CD3 + CD8 +) (per μL)	147	201–735
NK-cells (CD3-CD16/56 +) (per μL)	44	90–204
Hemoglobin (g/dL)	10.8	13.7–17.2
Transferrin (g/L)	1.2	2.15–3.65
Transferrin saturation (%)	80.9	16–45

**Fig. 2:**
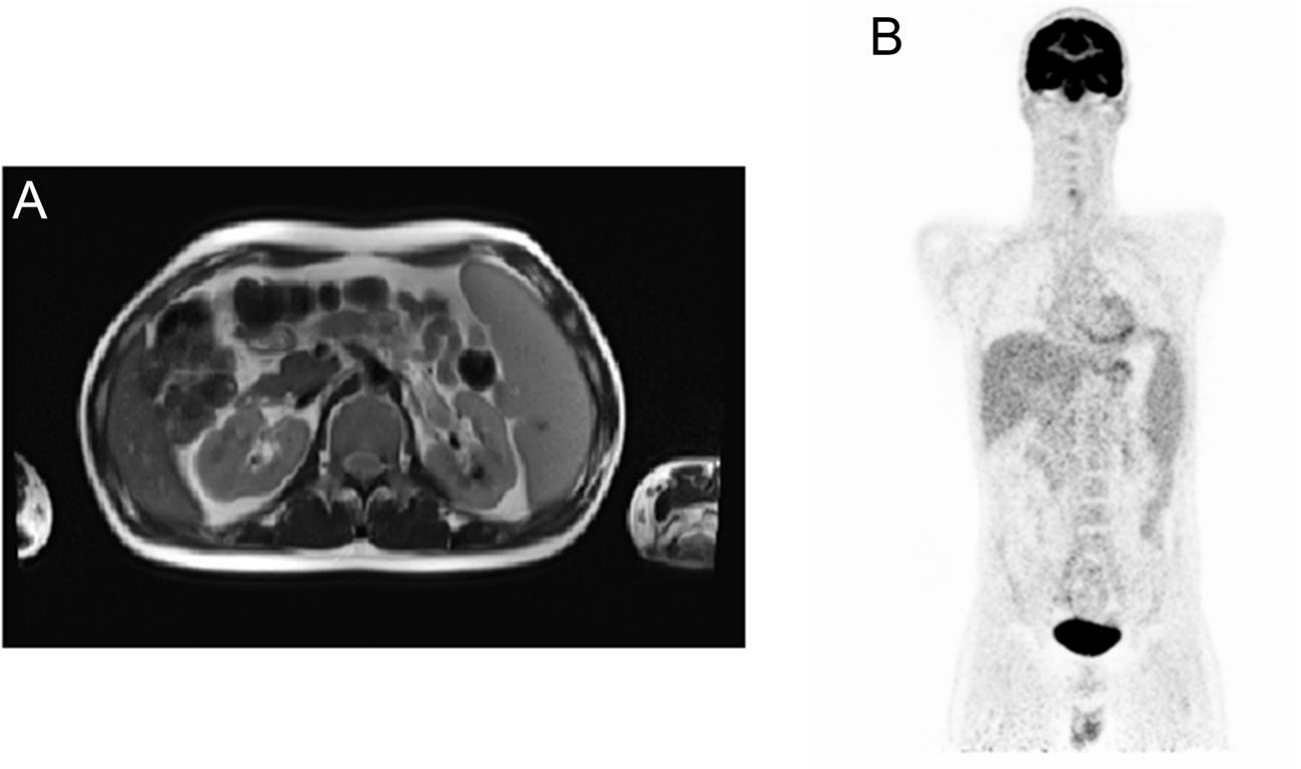
Maximum intensity projections of the fluorodeoxyglucose (FDG)-positron emission tomography/MRI (T2-HASTE) is showing a splenomegaly (**A**). No pathological FDG-uptake was detected (**B**)

Activity of chitotriosidase and the concentration of alpha-fetoprotein were measured to rule out Morbus Gaucher or ataxia telangiectasia. In the cerebrospinal fluid (CSF), cell counts and CSF-protein values were normal. Paraneoplastic antibodies and autoimmune antibodies in CSF were all negative. There was no measurable concentration of angiotensin-converting enzyme in CSF and normal concentration in serum. The CSF showed oligoclonal bands (type-1). Virological and bacteriological analyses were all negative, including severe acute respiratory syndrome coronavirus 2, herpes simplex virus, adenovirus, enterovirus, parechovirus, parvovirus, human immunodeficiency virus, JC virus, *T. whipplei*, *T. pallidum*, *B. burgdorferi* and *M. tuberculosis*. In neurovascular sonography, no pathologic flow profiles could be detected. Neuropsychological analysis demonstrated significant deficits in attention, processing speed, executive functions, learning ability, and retentiveness. In ophthalmologic examination, no uveitis or other inflammatory eye diseases could be identified. Abdominal sonography, chest X ray, and fluorodeoxyglucose-positron emission tomography/MRI of the whole body were unable to detect any other granulomatous inflammation apart from a splenomegaly (Fig. 2A, B). In abdominal sonography, liver parenchyma was slightly hyperechogenic as a sign of beginning steatosis.

Bone marrow puncture showed no acute hematological disease with normal cell counts but a slightly left shifted granulopoiesis. Erythropoiesis showed matured cells. In PAS-staining, some adjacent megakaryocytes and micromegakaryocytes were detected. In CD3- and CD-20 staining, some T- and B-cells were shown. No numeric or structural alterations in karyotype were found after chromosomal analysis.

Under suspicion of isolated granulomatous CNS inflammation, the patient received high-dose methylprednisolone for five consecutive days (1 g/day). In brain MRI, noduli with gadolinium enhancement were partially regressive. In contrast to imaging, the patient displayed a progressive cerebellar syndrome. Because of the progressive clinical disease course, plasma exchange was performed with subsequent intravenous immunoglobulin treatment (1 g per kilogram body weight for two consecutive days), with fever as a side effect, which led to rapid symptom improvement. CRP decreased to 2 mG per dL. At hospital discharge, the patient was able to walk without aid with no symptoms of cranial nerve impairment.

**Table 2:** Overview of performed medical diagnostics until the right diagnosis was confirmed

Relevant diagnostic tools	Main results
Lab analysis	Reduction of B- and T-lymphocytes, immunoglobulin (Ig)A, IgM, IgG2 and IgG4
	Elevation of ESR and CRP
	Normal values of chitotriosidase-activity and alpha-fetoprotein-concentration
Abdominal imaging	Splenomegaly, pancreatic lipomatosis and steatosis hepatis
MRI brain imaging	Paramedian brainstem infarction and granulomatous lesions
CSF analysis	Normal value for cell count and protein, no virological or bacterial infection, no paraneoplastic or autoimmune antibodies
Neurovascular sonography	Normal flow profiles
Neuropsychological analysis	Significant deficits in attention, processing speed, executive functions, learning ability and retentiveness
Ophthalmologic examination	Normal results
Chest X-Ray, abdominal sonography and fluorodeoxyglucose-positron emission tomography/MRI of the whole body	Splenomegaly, steatosis, no other granulomatous inflammation
Bone marrow puncture	Slightly left shifted granulopoiesis
Chromosomal analysis	No numeric or structural alterations in karyotype
Brain MRI (after beginning high-dose methylprednisolone)	Noduli with gadolinium enhancement partially regressive
Brain MRI (with new neurologic deficits five months after rehabilitation)	New paramedian brainstem infarction
ADA-2 enzyme activity	No activity found
Genetic analysis of ADA2 gene	Two pathologic variants were found (c.1358A > G and c.506G > A)

**Fig. 3:**
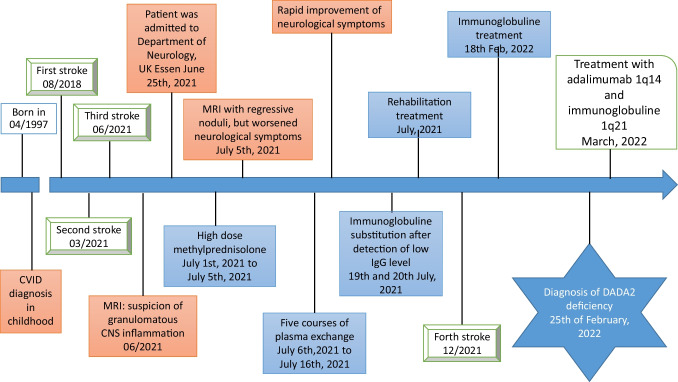
Timeline of key points in patients’ medical history. UK: University hospital

Five months after rehabilitation, the patient suffered from new hypesthesia on the left side of his face and his left hand. Brain imaging revealed a new paramedian brainstem infarction. CRP was elevated to 4.3 mG per dL again. ESR was at stable elevated values with 40 mm after 1 h and 74 mm after 2 h. The patient again received 60 g of intravenous immunoglobulins (0.75 g per kilogram body weight at day 1). Due to clinical course deficiency of adenosine deaminase 2 (DADA2) was suspected instead of CVID, with recurrent CNS vasculitis of the brainstem and the other observed clinical features (e.g., splenomegaly). After measurement, ADA-2 enzyme activity was found to be zero, confirming the diagnosis of DADA2. Two pathologic variants were found in genetic analysis of *ADA2* gene (c.1358A > G and c.506G > A). These variants were assessed as compound heterozygosity as causal reason for the underlying disease (Overview of relevant diagnostics in Table 2). Therefore, CVID was incorrectly diagnosed before. Maintenance therapy with subcutaneous immunoglobulins (200 g every 3 weeks) and adalimumab (40 mg every 2 weeks) was initiated and the patient has not suffered from any further disease episodes since then. No further strokes occurred. The patient’s neurological status improved until the last follow-up. Currently, he is able to do sports in a gym three times a week (Fig. 3).

## Discussion

DADA2 is a monogenic syndrome autosomal-recessive disease characterized by variable manifestations of systemic vasculitis, bone marrow failure, and immunodeficiency. DADA2 leads to a reduction of lymphocyte subsets and an increased concentration of tumor necrosis factor alpha (TNF-α) in plasma [1, 2]. Sharma et al. followed 33 cases of DADA2 [3]. Interestingly, 18 of these DADA2 patients developed at least one stroke. 25 patients were treated with TNF inhibitors, and all of them showed either clinical improvement or disease remission.

Not only ischemic but also hemorrhagic strokes are seen quite often in DADA2 patients. Hoffmann et al. reported of hemorrhagic strokes in 25% of a 24-patient cohort with biallelic germline mutations in the *ADA2* gene. The risk of hemorrhagic strokes was not significantly increased on anticoagulant or antiplatelet treatment. The authors concluded a high baseline risk of hemorrhagic stroke occurrence in DADA2 patients and suggest to avoid anticoagulant or antiplatelet treatment in this population [4]. In our case, we have treated the patient with enoxaparin in antithrombotic dosage for a couple of days because he had a high risk of thrombosis due to his neurological deficits and immobility. After confirmation of DADA2 and improvement of neurological symptoms, we stopped the enoxaparin therapy immediately. It is not known, if the patient also received enoxaparin during his first strokes in August 2018 and March 2021, but he was not immobile and there was no other indication for antithrombotic therapy at that time.

Ombrello et al. described 15 patients with DADA2 who were suffering from strokes. After initiating a therapy with TNF-α inhibitors, no further strokes occurred in these patients while recurrent strokes occurred in the patients receiving intravenous immunoglobulines and steroids prior to TNF initiation [2]. In our case, the patient’s symptoms worsened during glucocorticoid therapy, the reason why we escalated the treatment. A further stroke occurred after initiating an intravenous immunoglobulin therapy, but after adding adalimumab no further strokes occurred. Therefore, the treatment schedule was not adjusted anymore until the last follow-up.

CVID is the most prevalent symptomatic primary immune deficiency. Hallmarks of CVID are hypogammaglobulinemia and recurrent infections. The etiology of CVID is mostly unknown, with no universally accepted definition. CVID is a diagnosis of exclusion, based on European Society for Immunodeficiencies criteria [5, 6] and was suggested in our patient before DADA2 was confirmed. Different genetic reasons are involved in the development of disorders associated with CVID, particularly monogenetic aberrations and epigenetic modifications [6]. The transmembrane activator calcium modulator and cyclophilin ligand interactor (TACI) and B-cell activating factor (BAFF) are different members of the TNF superfamily for example. The BAFF receptor has been described as a potential genetic abnormality in CVID. BAFF-R and TACI are associated with B-cell development and activation [6–8]. As described, TNF has also been shown to be involved in DADA2 pathogenesis. In CVID, severe B-cell deficiency leads to hypogammaglobulinemia. A deficiency in frequency and function of T-cells is also described in CVID. The defects and deficiencies lead to alternate B-cell–T-cell interactions and finally to repetitive bacterial, viral, and fungal infections [9].

We describe a patient with granulomatous CNS inflammation and cerebellar dysfunction caused by DADA2 deficiency. Initially, CVID was suggested and the patient suffered from further strokes due to insufficient therapy approach. The simultaneously impaired and hyperactive immune response led to an individual therapeutic regimen that included immunosuppressive and immunomodulatory treatment [10, 11]. Stroke related to CVID has been described in context with immunoglobulin treatment but also due to concomitant CVID with Moyamoya disease and Takayasu arteritis [12, 13]. Contrast-enhancing lesions of the brain or spinal cord are known to be the most common findings on MRI in patients with CVID and neurological manifestation. Next to CNS granulomas, meningoencephalomyelitis or signs of CNS vasculitis are described as specific MRI findings. There is no evidence of the best immunomodulatory therapy in the literature for CVID patients with CNS manifestation. That may be the result of the fact that, as a clinical diagnosis, the underpinnings of disease are different for most of the cases presenting with CNS manifestations, thus a unified treatment is highly unlikely. An individual immunosuppressive therapy with azathioprine, high-dose steroids, cyclophosphamide, adalimumab, cyclosporin, or hematopoietic stem cell transplantation have been suggested [14].

## Conclusion

We present the case of a young adult with a delayed diagnosis of DADA2 and recurrent stroke due to vasculitis. Patients often experience a multitude of medical diagnostics during a disabling disease course. ADA2 deficiency is a rare stroke etiology, but should be suspected especially in young stroke patients with lab abnormalities suggesting CVID. Early diagnosis allows for an effective treatment with TNF-alpha inhibitor to prevent strokes. Intravenous immunoglobulins alone are not sufficient enough to prevent further strokes. In patients who present atypical features of an established diagnosis seeking for alternative explanations should be continued until the correct diagnosis is ensured.

## Data Availability

The original clinical datasets generated during the case are available from the corresponding author on reasonable request.
